# Anodic bonding of mid-infrared transparent germanate glasses for high pressure - high temperature microfluidic applications

**DOI:** 10.1080/14686996.2019.1702861

**Published:** 2020-01-13

**Authors:** Julien Ari, Geoffrey Louvet, Yannick Ledemi, Fabrice Célarié, Sandy Morais, Bruno Bureau, Samuel Marre, Virginie Nazabal, Younès Messaddeq

**Affiliations:** a Centre d’Optique, Photonique et Laser (COPL), Université Laval, Québec (QC), Canada; b Equipe Verres & Céramiques - Institut des Sciences Chimiques de Rennes (ISCR), UMR-CNRS 6226, Université de Rennes 1, Rennes, France; c Département Mécanique & Verres, Institut de Physique de Rennes (IPR), UMR-CNRS 6251, Université de Rennes 1, Rennes, France; d Institut de Chimie de la Matière Condensée de Bordeaux (ICMCB), UMR-CNRS 5026, Université de Bordeaux, Bordeaux, France

**Keywords:** Germanate glass, mid-infrared, anodic bonding, high pressure/high temperature, microfluidics, 107 Glass and ceramic materials, 204 Optics / Optical applications, 300 Processing / Synthesis and Recycling, 208 Sensors and actuators, 505 Optical / Molecular spectroscopy

## Abstract

High pressure/high-temperature microreactors based on silicon-Pyrex® microfabrication technologies have attracted increasing interest in various applications providing optical access in high-pressure flow processes. However, they cannot be coupled to infrared spectroscopy due to the limited optical transparency (up to ~2.7 μm in the infrared region) of the Pyrex® glass substrate employed in the microreactor fabrication. To address this limitation, the alternative approach proposed in this work consists in replacing the Pyrex® glass in the microreactor by a mid-infrared transparent glass with thermal and mechanical properties as close as possible or even better to those of the Pyrex®, including its ability for silicon-wafers coupling by the anodic bonding process. Glasses based on germanate GeO_2_, known for their excellent transmission in the mid-infrared range and thermal/thermo-mechanical properties, have been thus evaluated and developed for this purpose. The optical, mechanical, thermal and electrical conductivity properties of adapted glass compositions belonging to five vitreous systems have been systemically investigated. The glass composition 70GeO_2_-15Al_2_O_3_-10La_2_O_3_-5Na_2_O (mol.%) was defined as the best candidate and produced in large plates of 50 mm diameter and 1 mm thickness. Anodic bonding tests with Si-wafers have been then successfully conducted, paving the way for the development of fully mid-infrared transparent silicon-glass microreactors.

## Graphical abstract

**Figure:**
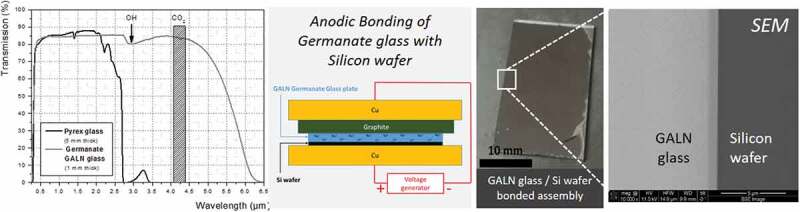

## 1. Introduction

The wide development of microfluidic applications in chemistry [1,2], biology [3,4], and materials science [5] has opened avenues towards *in situ* fast screening capability and (bio) chemical detection labelling at microscale. Microfluidic systems are increasingly used for monitoring the evolution of chemical synthesis processes, due to their low reagent consumption and faster equilibrium times, allowing a better control of the process and less risky manipulation thanks to smaller volumes. This latter advantage is of particular importance when considering high-pressure processes at microscale. Indeed, over the last 10 years, high pressure/high temperature (HP/HT) microfluidics has gained increasing interest, being developed and used for various applications including catalysis [6], nanomaterials synthesis [7,8] or thermodynamics data determination [9]. Another recent application concerns the development of the so-called ‘geological labs on chip’ or ‘micromodels’ for investigating pore-scale-related topics such as carbon capture & storage (CCS) in deep saline aquifers or enhanced oil recovery by mimicking on chip the conditions (temperature, pressure, permeability) of geological reservoirs [10–12].

**Figure 1.**
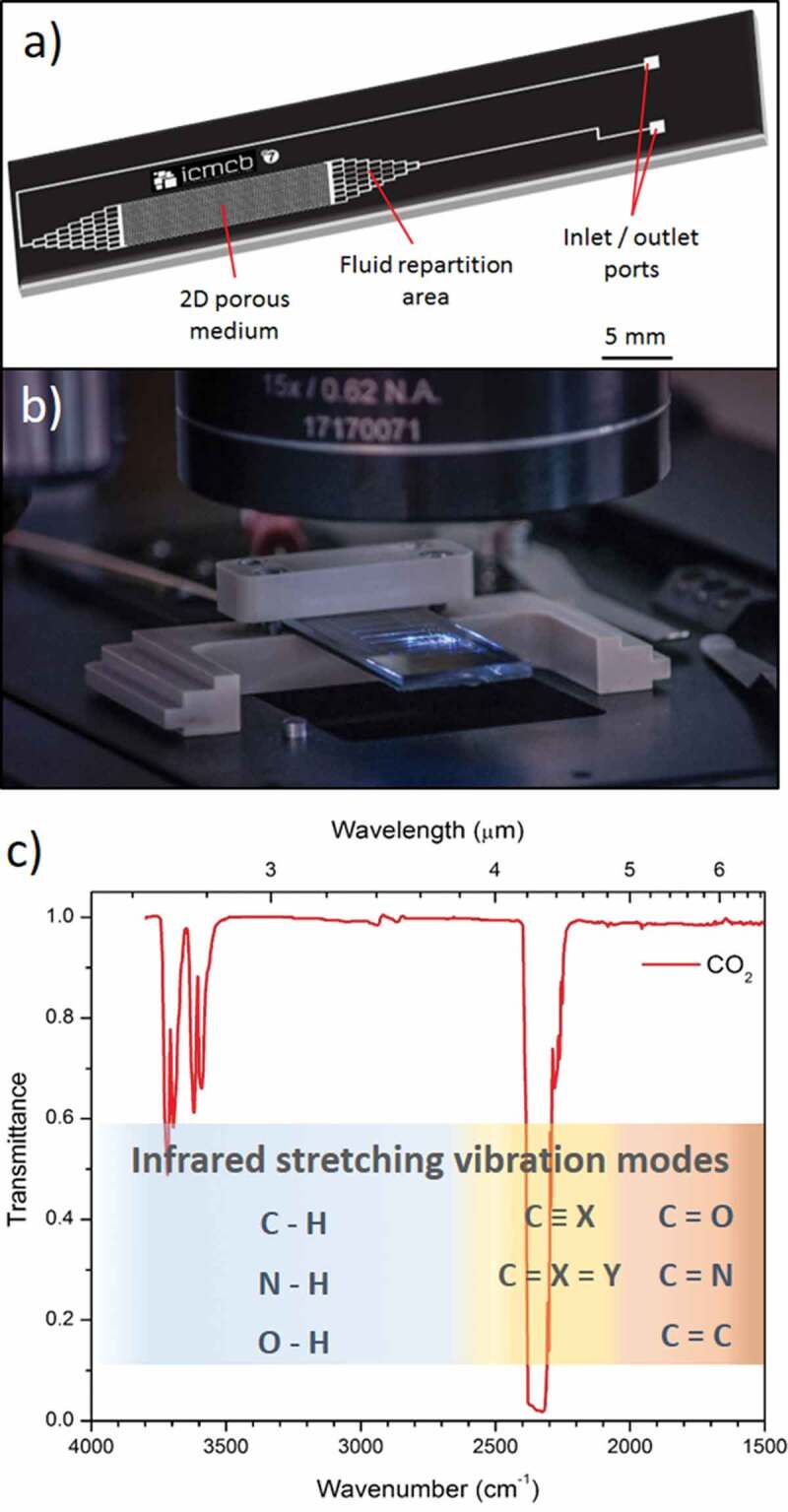
(a) Scheme of a silicon-Pyrex® glass microreactor and (b) Photograph of a microreactor under an objective microscope. (c) Infrared transmittance spectrum in the 2–6 µm wavelength range (4000–1500 cm^−1^ wavenumber range) of CO_2_. The main molecular stretching vibration modes observable in this range are also presented

To manufacture such kind of pressure-resistant microdevices, HP/HT microreactors have beneficiated from recent scientific advances on different types of substrates (glass-glass or silicon-Pyrex®) [13–16]. In particular, silicon-based devices, which allow inscribing microchannels by photolithography, while taking advantage of the good thermo-mechanical properties of silicon have been largely employed [12,17,18]. The fabrication of Si-glass microdevices is well developed and convenient. Once the silicon substrate is patterned and further etched (wet etching or deep reactive ion etching [19]), the assembly with the glass is realized using the well-known anodic bonding process. Such a bonding process is totally compatible with HP/HT applications, since temperature of ~400°C and pressure up to 30 MPa were already reported for the use of such microreactors [17]. Given the great potential of such microsystems, the implementation of *in situ* characterization techniques into these devices is highly desirable in order to monitor the evolution of various processes or to detect specific (bio)chemical species qualitatively and quantitatively. In this context, optical spectroscopy techniques have already been widely coupled to microfluidic devices including Raman [20,21] and UV-Visible, as illustrated in Figure 1(a,b) [22]. Recently, mid-infrared (mid-IR) spectroscopy has also been implemented inside microreactors for analysing complex media, but always for conditions close to conventional pressures and temperatures [23–26]. To achieve high-pressure capability, F. Starecki et al. have proposed a system based on a chalcogenide fibre doped with Dy^3+^ ions embedded in a Si-Pyrex® microreactor to study *in situ* the CO_2_ behaviour through the microchannel [27]. The dysprosium ion can generate luminescence at 4.3 µm under an appropriate pumping wavelength, which allows the detection of the carbon dioxide absorption band at the same wavelength [28]. However, this approach has two main limitations. First, the relatively weak mechanical resistance of the system limits the pressure and temperature ranges for operation. A maximum pressure of 70 bars was reported. Then, only single-point detection can be performed inside the microreactor, whereas achieving imaging of the whole reactor could provide additional advantages for process monitoring.

Additionally, using conventional borosilicate glasses such as commercial Pyrex® (80.6%SiO_2_ – 13.0%B_2_O_3_ – 4.2%Na_2_O – 2.2%Al_2_O_3_ (mol.%)) to produce HP/HT microreactors limits inevitably the transparency windows of the device to 2.7 μm in the infrared range. This, therefore, impedes their coupling with infrared/Raman spectroscopic techniques and for instance the detection of the main molecular stretching vibration modes, including CO_2_ (see Figure 1(c)). Mid-IR transparent calcium fluoride windows (transparent from 0.2 to about 10 µm depending on their thickness) were already integrated in microreactors. However, investigating microscale processes in harsh conditions with such systems is hindered by the weak mechanical properties of CaF_2_ related to its brittle nature. Thus, developing alternative mid-IR transparent materials – capable of (i) withstanding relatively high pressure and temperature and (ii) being compatible for bonding with Si wafer – is of first interest to produce HP/HT mid-IR microreactors [29]. To this end, germanium oxide-based glasses appear as promising candidates in terms of mid-IR transmission, thermal and mechanical properties [30–34]. Moreover, a careful design of the germanate glass composition may also allow to employ the anodic bonding process, which has demonstrated its strong efficiency for assembling Si-Pyrex® HP/HT microdevices [35,36].

Based on the above-mentioned consideration, the objective of this work is to develop a germanate glass displaying a wide transparency ranging from the UV-visible to the mid-IR (~5 μm), compatible with the anodic bonding process and which can be used for both visible and mid-IR *in situ* characterization in HP/HT microreactors. The preparation and shaping of five different compositions of germanate glasses are demonstrated. Their optical, thermal and thermo-mechanical properties have been characterized. Their electrical conductivity as a function of temperature as well as their Vickers hardness, Poisson’s ratio, density and elastic modulus at room temperature have been also determined and compared with those of commercial Pyrex® glass. Finally, anodic bonding tests on silicon wafers have been conducted from a selected germanate glass composition and compared with those achieved with Pyrex® glass substrates.

## 2. Materials and methods

### 2.1. Germanate glass compositions and synthesis

The glasses are prepared by the conventional melting-cast method. First, the starting elements GeO_2_ (5N), Ga_2_O_3_ (3N), La_2_O_3_ (3N), Al_2_O_3_ (3N), CaCO_3_ (3N), BaCO_3_ (3N), Na_2_CO_3_ (5N), CaF_2_ (3N) and BaF_2_ (2N) are weighted and mixed following the molar compositions presented in Table 1, and adapted from reported literature [37–41]. Then, the powders are introduced in a platinum crucible and pre-heated in an induction furnace under 2 L/min nitrogen flow at 500°C (15 min) and 1200°C (15 min) for dehydration and decarbonation processes, respectively. The melting/fining step is achieved at 1400–1500°C for 90 min depending on the composition. After that, the melted glass is poured in a stainless steel mould preheated at 40°C below the glass transition temperature (T_g_), annealed for 5 h at the same temperature and slowly cooled down to an ambient temperature at 1°C/min. Finally, the glass samples are cut and polished in 10 mm-diameter and 5 mm-thick cylinders to perform all the physico-chemical characterizations.

**Table 1.** Chemical compositions of the glasses under study

Sample Label	Glass composition (mol.%)	Adapted from:
GACCN	70GeO_2_-10Al_2_O_3_-10CaO-5CaF_2_-5Na_2_O	[37]
GGBBN	65GeO_2_-15Ga_2_O_3_-10BaO-5BaF_2_-5Na_2_O	[38]
GGCN	80GeO_2_-10Ga_2_O_3_-5CaO-5Na_2_O	[39]
GGLN	55GeO_2_-30Ga_2_O_3_-10La_2_O_3_-5Na_2_O	[40]
GALN	70GeO_2_-15Al_2_O_3_-10La_2_O_3_-5Na_2_O	[41]
Pyrex®	80.6SiO_2_–13.0B_2_O_3_–4.2Na_2_O–2.2Al_2_O_3_	Corning Pyrex 7740

The same procedure is used to prepare the 50 mm-diameter glass disks of 1 mm thickness for the anodic bonding tests, except that the glass melt is pressed between two stainless steel plates pre-heated at T_g_ - 40°C. Such a procedure was implemented because of the high viscosity of the glass melts at the casting temperature, which complicated the formation of thin samples. The glass plates are polished until 1 µm finishing to ensure a good contact with the silicon wafer during the anodic bonding.

### 2.2. Optical and thermal characterizations

The optical transmission spectra have been recorded on 5 mm-thick bulk samples using two spectrophotometers for the UV-visible and the mid-IR regions. The first one is an UV-vis/NIR Agilent Technology Cary 5000 and the second one is a FT-IR/FIR Frontier Perkin Elmer.

The onset glass transition T_g_ and crystallization T_x_ temperatures of all glasses have been determined by differential scanning calorimetry (DSC). Measurements were performed using a Netzsch DSC 404 F3 Pegasus on approximately 25 mg glass pieces in a platinum pan at a heating rate of 10°C/min up to 1000°C.

The thermal expansion coefficient (TEC) has been determined using a Netzsch TMA 402 F1 Hyperion instrument on 10 mm-diameter and 5 mm-thick cylindrical samples, at a heating rate of 5°C/min, an ending temperature of T_g_ – 50°C and a pushrod force of 0.02 N. The TEC values were obtained from the curve slope in the 100–400°C temperature range for all the samples.

### 2.3. Electrical conductivity measurement

Electrical characterizations have been performed on the same cylindrical samples, using a 1260 Solartron impedance analyser with an applied voltage of 1 V. Measurements are realized in AC current at 1 MHz frequency, using two platinum electrodes into a Probostat TM Norecs probe sample holder placed in a tubular furnace. Temperature dependence measurements are realized under ambient atmosphere from 50°C to 500–650°C depending on the sample's glass transition temperature.

### 2.4. Mechanical properties and density measurement

Vickers hardness at ambient temperature is measured by the indentation technique at 1 N using a Fisherscope H100C equipped with a diamond pyramid with a square base indenter. The charge application time and the loading rate are 20 s and 50 mN/s, respectively. Ultrasonic echography (USE) method with 10 MHz piezoelectric transducers is used to determine the Poisson’s ratio (υ), the Young (E) and Coulomb (G) moduli at room temperature. A coupling gel is used to optimize the contact between the sample and the transducers. These measurements have been performed on cylindrical samples of equal dimensions. The elastic moduli (E and G) as a function of the temperature are measured with a resonant frequency damping analysis (RFDA). This last measurement has been performed on glass plate samples suspended on Pt/Rh wires placed in a classical resistive furnace, from room temperature to T_g_ + 50°C. This method consists in an impulse excitation technique where the sample is periodically impacted with a ceramic spike. The vibration frequency of the sample is then recorded by a microphone. More details about the calculation method are given elsewhere in the literature [42–44].

The density has been measured by the Archimedes’ method in absolute ethanol at room temperature using a Mettler Toledo XS64 analytical balance.

### 2.5. Germanate glass substrate fabrication, cleaning and pre-bonding

The anodic bonding process has been performed with glass plates of 5 cm diameter and 1 mm thickness. After polishing, the surface of the samples is analysed with a Zygo MetroPro OMP-0347K optical surface state interferometer. Then, silicon wafers are cleaned successively with HF to remove the silicon oxide passive layer, and with a piranha solution to eliminate any residual organic compounds at the surface. Then, they are rinsed successively with deionised water, acetone and ethanol. Glass plates are directly washed with deionised water, acetone and ethanol. Finally, both plates are dried and put in contact together for a pre-bonding before starting the process.

Silicon wafers (p type) of 7.62 cm (3 inches) diameter with a resistivity between 1 and 10 ohm.cm^−1^ are used. These wafers are 1 mm thick, double-side polished and <100> oriented. A strict and rigorous cleaning process for both the silicon wafers and the glass plates has been implemented and conducted entirely in a cleaning room (class 1000).

### 2.6. Anodic bonding setup

**Figure 2.**
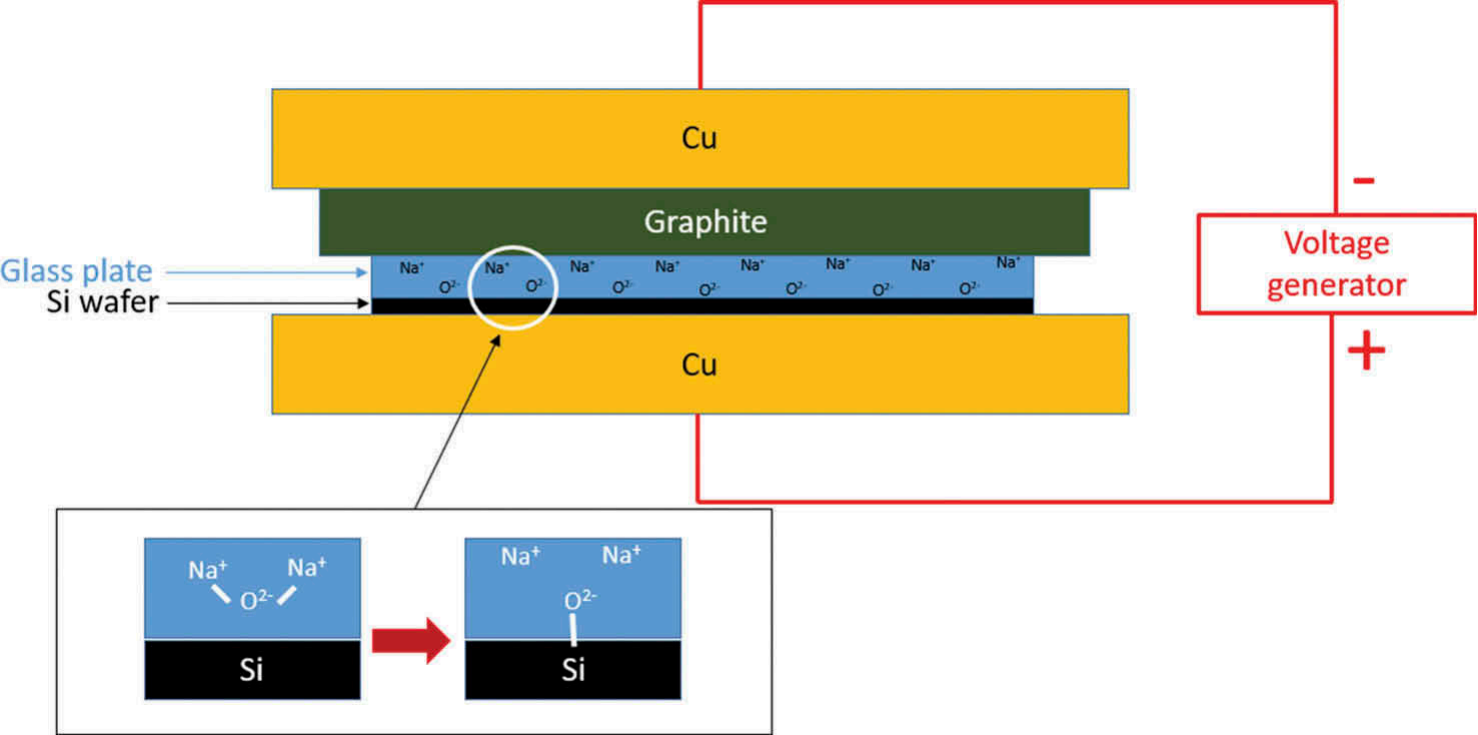
Schematic view of the anodic bonding setup

The bonding is performed in a Nabertherm furnace at a temperature between 250°C and 375°C depending on the glasses' composition and their T_g_. A voltage in the range of 250–1750 V is applied with a Hewlett-Packard 6110A DC power supply. A schematic view of the setup is presented in Figure 2.

This setup is similar to what is already used for the fabrication of Si-Pyrex® microsystem. It is composed of two copper electrodes (of 5.3 kg each) and a graphite plate to protect the glass substrate while allowing the current to go through the electrodes. Considering the top electrode weight and the sample dimensions, the pressure applied to the glass during the bonding process is calculated to be 26 500 Pa. All the components are placed in the furnace to ensure their homogeneous heating and to avoid the formation of undesired thermal gradients between electrodes because of their different thermal conductivities. The three main parameters which determine the bonding quality are the temperature, the voltage and the time of exposition [45]. These values depend on the glass composition.

### 2.7. Bonding parameters

The bonding parameters, including the temperature and the voltage, have been determined after multiple preliminary tests at various temperatures and voltages. During the optimization of the anodic bonding process, the set of temperature and voltage for which the best results were achieved in terms of bonding (adherence, transparency of the glass substrate preserved, no cracks of the glass substrate, etc.) were selected.

The optimum bonding temperatures and voltages were therefore fixed at 250°C and 375°C (with a heating rate of 10°C/min), and 1250 and 1550 V, respectively, for the Pyrex® and the germanate glass. Voltage, time and current parameters for both Pyrex® and germanate glasses are listed in Table 2.

**Table 2.** Bonding parameters for the: (a) Pyrex® glass. (b) Germanate glass

(a) Pyrex® glass (250°C)			(b) Germanate glass (375°C)		
Voltage [V]	Time [min]	Current [mA]	Voltage [V]	Time [min]	Current [mA]
250	1	0.5	250	1	0.5
450	1	0.9	450	1	0.9
550	1	1.0	650	1	1.2
650	1	1.1	750	1	1.4
750	2	1.4	850	2	1.6
850	5	1.6	950	2	1.8
950	5	1.9	1050	2	2.0
1050	10	2.0	1150	5	2.2
1150	10	2.2	1250	5	2.4
1250	15	2.5	1350	10	2.6
250	10	0.5	1450	10	2.9
			1550	15	3.0
			250	5	0.5

The voltage is applied gradually, up to 1250 and 1550 V for, respectively, the Pyrex® (Table 2(a)) and the germanate glass (Table 2(b)). First, low voltages are applied systematically for short periods of typically 1 to 2 min. Then, periods are increased up to 5–15 min at high voltage to ensure the sodium cations' migration and to maximize the bonding quality. A last step at 250 V is performed before turning off the DC voltage generator. At the end of the process, the temperature is slowly decreased to room temperature by simply switching off the furnace.

### 2.8. Scanning electron microscopy

To investigate the quality and the migration of species at the interface between the glass and the silicon wafer, scanning electron microscopy (SEM) analyses using a Quanta 3D-FEG instrument equipped with an energy dispersive X-ray (EDAX) spectrometer were carried out. First, the Si-glass assemblies were cut with a precision dicing saw to get access to the bonded region. Secondary electron imaging mode was used to obtain high-resolution images, whereas backscattered electron mode was employed to assess information related to the elemental distribution (contrast) with the samples. Energy dispersive analyses have been performed by scanning lines crossing the interface from the glass to the silicon wafer.

## 3. Results and discussion

### 3.1. Germanate glasses' characterization

The selection of the five germanate glass compositions was directed according to three main criteria besides their transparency in the mid-IR range (up to 5–6 µm). First, their glass transition temperature has to be similar or higher than that of Pyrex®, i.e. ≥550–560°C. This criterion, therefore, excludes the well-known lead-germanate glasses and imposes the gallo-germanate or alumino-germanate based systems. Second, as the role played by the mobile sodium cations was demonstrated in the anodic bonding process of Pyrex® glass with silicon, a similar concentration of sodium oxide (i.e. 5 mol.%) is added to the germanate glass compositions. Last, the addition of alkali-earth (calcium or barium) oxides and fluorides is explored. Alkali-earth oxides act as network modifier, decreasing the glass viscosity at high temperature, where a small amount of fluoride is added in order to decrease the OH content within the glass. The five compositions studied here are listed in Table 1.

#### 3.1.1. Optical transmission

**Figure 3.**
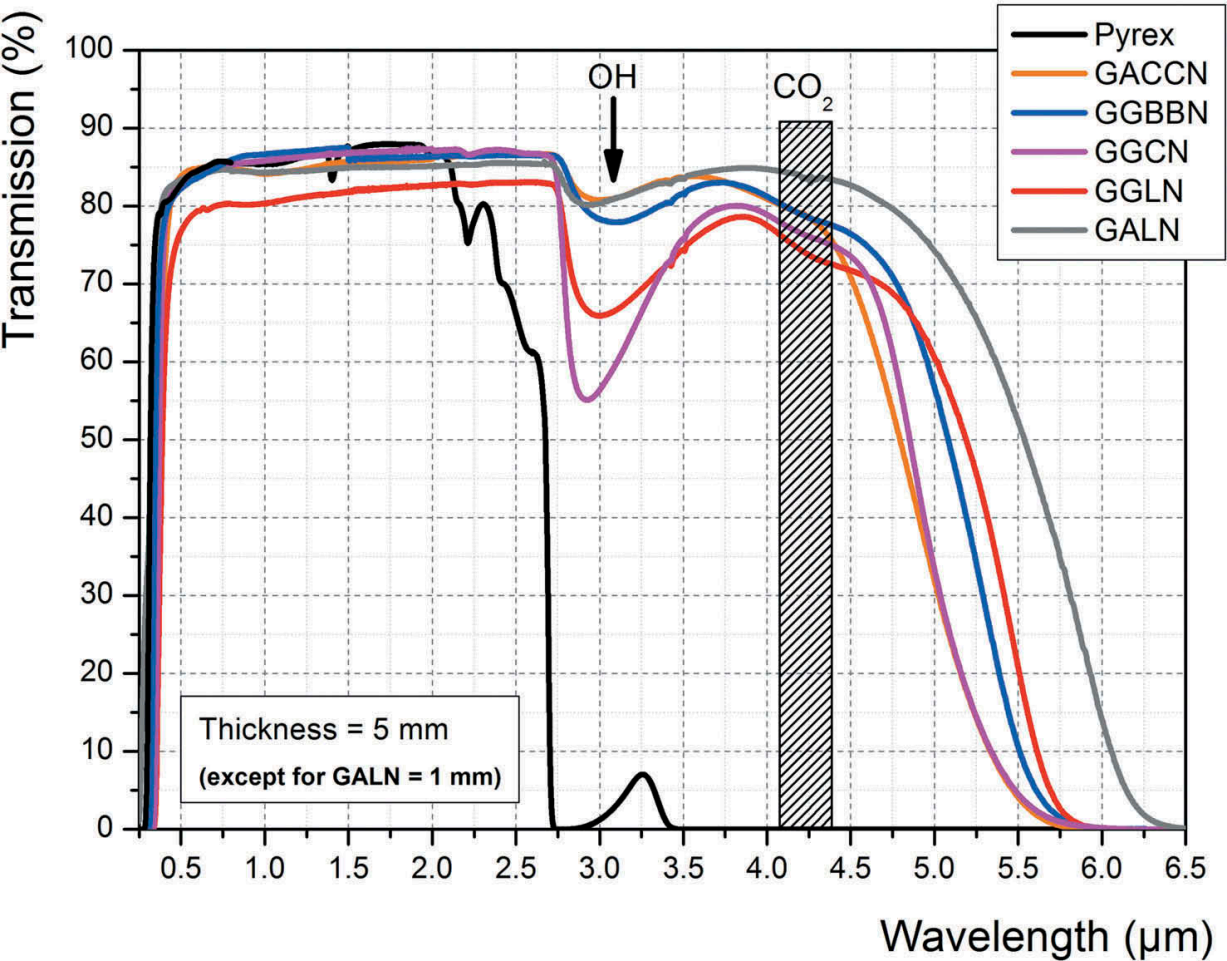
Optical transmission spectra of the five germanate glasses under study compared to that of a commercial Pyrex® glass. Sample thickness is 5 mm except for the GALN glass for which thickness is 1 mm (GACCN: 70GeO_2_-10Al_2_O_3_-10CaO-5CaF_2_-5Na_2_O; GGBBN: 65GeO_2_-15Ga_2_O_3_-10BaO-5BaF_2_-5Na_2_O; GGCN: 80GeO_2_-10Ga_2_O_3_-5CaO-5Na_2_O; GGLN: 55GeO_2_-30Ga_2_O_3_-10La_2_O_3_-5Na_2_O; GALN: 70GeO_2_-15Al_2_O_3_-10La_2_O_3_-5Na_2_O, mol.%)

Figure 3 presents the optical transmission spectra recorded on the five germanate glasses' compositions prepared in this work, compared to that recorded on a Pyrex® commercial sample. One can see in Figure 3 that the 5 mm-thick Pyrex® sample becomes opaque from around 2.7 µm, whereas all the germanate glasses transmit up to 5.5–5.7 µm before reaching 0% of transmission. Note that the transmission spectra presented for GALN composition were recorded on a 1-mm-thick glass sample, illustrating the role of material thickness in the transmission at longer infrared wavelengths. In the transmission spectra of these GeO_2_-based glasses, an absorption band is observed at 3.0 µm related to their residual OH content. Further purification to reduce the OH absorption band can be useful but is not critical at this step to demonstrate the potential of using such glasses for HP/HT microfluidic applications. Thus, all these germanate glasses can be potentially used for the targeted application from an optical transparency point of view.

#### 3.1.2. Thermal expansion and characteristic temperatures

Table 3 summarizes the thermal properties measured for the five germanate glasses along with the one of the Pyrex® glass. The five germanate glasses prepared possess a TEC ranging between 6.9 and 8.7 × 10^−6^ K^−1^, which is relatively higher than that of the Pyrex® (3.3x10^−6^ K^−1^). It is also worth reminding that the silicon’s TEC is 2.6 × 10^−6^ K^−1^. Such large TEC values measured for the germanate glasses can, therefore, be a limiting factor for their utilization in HP/HT microfluidic applications because a thermal stress could be induced at the interface between the glass and the silicon wafer during the anodic bonding process. In particular, a too large TEC difference between the germanate glass substrate and the Si-wafer may result in microreactor fracture during the post-bonding cooling stage.

**Table 3.** Thermal expansion coefficient (TEC), glass transition temperature (T_g_) and thermal stability against crystallization (T_x_-T_g_) measured for all the glasses under study

Glass sample	TEC [±0.1x10^−6^ K^−1^]	T_g_ [±2°C]	T_x_ – T_g_ [±4°C]
Pyrex®	3.3	560	*
GACCN	8.7	568	157
GGBBN	8.8	552	162
GGCN	6.7	550	*
GGLN	6.5	676	130
GALN	6.9	690	216

With respect to the glass transition temperature, all the germanate glasses possess a higher T_g_ than Pyrex®’s. The highest T_g_ are observed for the alumino-germanate 70GeO_2_-15Al_2_O_3_-10La_2_O_3_-5Na_2_O (GALN) and 55GeO_2_-30Ga_2_O_3_-10La_2_O_3_-5Na_2_O (GGLN) glasses. It can be explained by an high average coordination number compared to other compositions owing to their lower contents in alkali-earth and alkali oxides to introduce lanthanum oxides and aluminium oxides or gallium oxides in greater proportion [46,47]. Overall, adding more GeO_2_ glass-former and to a less extent both glass-formers/-intermediates (Ga_2_O_3_, Al_2_O_3_La_2_O_3_) will increase the concentration of bridging oxygens and therefore reinforce the glassy network [48]. This results in an increase of the glass transition temperature and a decrease of its thermal expansion coefficient. So these glasses can be used at higher temperatures than Pyrex without any softening deformations.

The thermal stability against crystallization criterion T_x_ - T_g_ of such glasses is well above 100°C for all the studied compositions, denoting an excellent glass-forming ability. The pouring of large glass pieces is therefore expected to be facilitated. Moreover, the glass shaping and/or processing at temperatures close to its T_g_ or below should not induce any crystallization, which is of first importance not only for the targeted application, but also for the anodic bonding process. During the latter step, the glass plate in contact with the silicon wafer is indeed heated below Tg in addition to the DC voltage application.

Regarding the thermal properties, the GALN and GGLN glasses show respectively the lowest and highest values in terms of TEC, T_g_ and T_x_ - T_g_ for the targeted application. That means they can be potentially used at a higher temperature than 560°C (= T_g_ of Pyrex®). On the other hand, although they exhibit the lower thermal expansion coefficient (ranging between 6.5 and 6.9 × 10^−6^ K^−1^) among the studied germanate glasses, both the GALN and GGLN will be more sensitive to thermal shocks (if heating rates are too high for instance) than Pyrex® glass.

#### 3.1.3. Electrical conductivity

**Figure 4.**
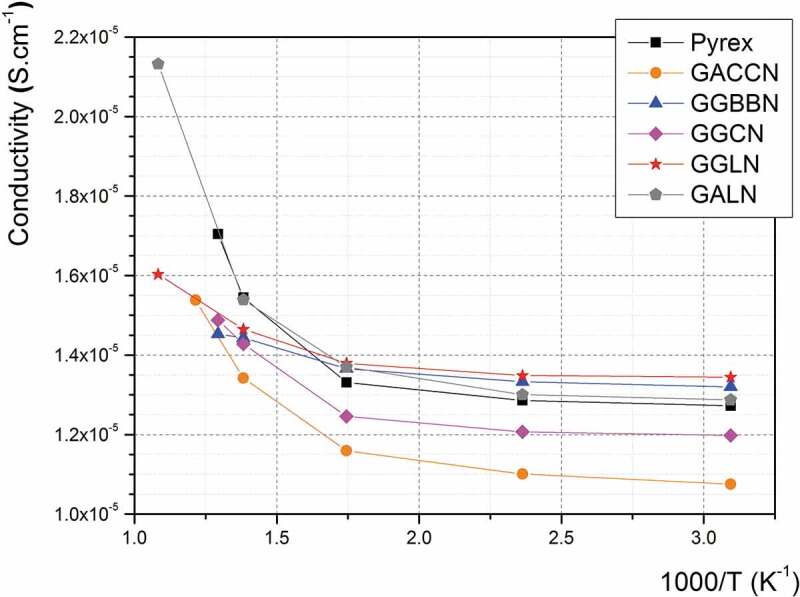
Calculated electrical conductivity as a function of the reciprocal temperature measured on the five germanate glasses under study (1 MHz AC frequency, 1 V applied), in comparison with the Pyrex®’s values (GACCN: 70GeO_2_-10Al_2_O3-10CaO-5CaF_2_-5Na_2_O; GGBBN: 65GeO_2_-15Ga_2_O_3_-10BaO-5BaF_2_-5Na_2_O; GGCN: 80GeO_2_-10Ga_2_O_3_-5CaO-5Na_2_O; GGLN: 55GeO_2_-30Ga_2_O_3_-10La_2_O_3_-5Na_2_O; GALN: 70GeO_2_-15Al_2_O_3_-10La_2_O_3_-5Na_2_O, mol.%)

Prior to initiating the anodic bonding experiments, the electrical conductivity of these glasses has been investigated and compared with that of Pyrex®. Indeed, the electrical conductivity in such glasses is mainly governed by small ions motion (e.g. Na^+^ ions [49,50]) which are also involved in the anodic bonding process [35,36]. Assessing the ionic conductivity in these materials can, therefore, bring relevant information about their potential for the anodic bonding process. The measured electrical conductivities are presented in Figure 4 for each glass composition as a function of reciprocal temperature.

The calculated AC electrical conductivity measured on all the germanate glasses is ranging between 1.07 × 10^−5^ S.cm^−1^ and 1.34 × 10^−5^ S.cm^−1^ at room temperature and between 1.45 × 10^−5^ S.cm^−1^ and 2.14 × 10^−5^ S.cm^−1^ at a temperature close to their respective T_g_. The increase of conductivity measured with increasing the temperature denotes an ionic conduction mechanism, as expected. We assume that this phenomenon is mainly governed by the mobility of the Na^+^ ions, which are the smaller ions in all the investigated glassy network, as already reported elsewhere [51,52]. Furthermore, one can above all note in Figure 4 that the Pyrex® glass, whose anodic bonding with silicon wafer has already been demonstrated [53], exhibits a similar ionic conductivity in the temperature range of interest. Considering that the conductivity mechanisms in the Pyrex® and the studied glasses are similar (both related to Na^+^ mobility), these results support the potential of using these germanate glass compositions for anodic bonding. Figure 4 also shows that electrical conductivities are slightly increasing between 50°C and 150°C (corresponding, respectively, to 323 and 423 K). Then, they are following a fast increase for temperatures between 300°C (573 K) and T_g_ (460-560°C corresponding to 733–833 K depending on the glass composition).

In particular, the GALN glass exhibits conductivity values very close to the Pyrex® ones from room temperature to 500°C, which is the highest temperature of operation in a HP/HT system for the latter (due to the Pyrex® T_g_ = 560°C). For temperatures above 500°C, the electrical conductivity of the GALN glass, whose T_g_ is 690°C, is increasing quickly, up to 2.14 × 10^−5^ S.cm^−1^ at 650°C. According to the experimental results presented in Figure 4, the GALN alumino-germanate and the Pyrex® glasses show a similar electrical conductivity behaviour, governed by the Na^+^ ions mobility in both matrices [54–56]. Furthermore, the GALN glass can be potentially employed at a higher temperature than the Pyrex® thanks to its higher T_g_. This is why the 70GeO_2_-15Al_2_O_3_-10La_2_O_3_-5Na_2_O (GALN) glass was determined as the best candidate among the germanate glasses studied here for the fabrication of HP/HT microreactors operating in the mid-IR range.

#### 3.1.4. Mechanical behaviour – Vickers hardness, elastic modulus and poisson’s ratio

**Figure 5.**
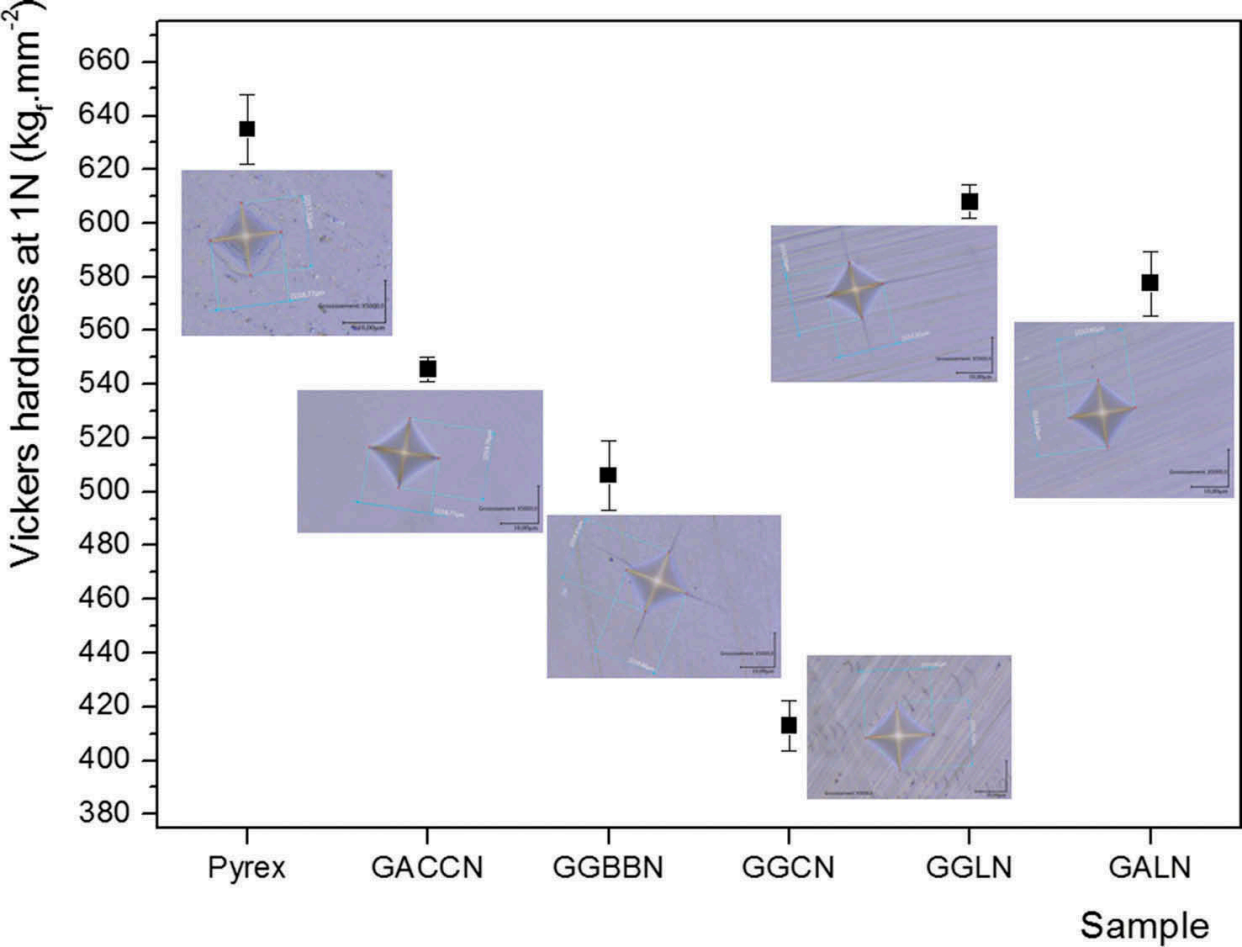
Vickers hardness and photographs of the indentation prints for the five germanate glasses under study compared with the Pyrex® (GACCN: 70GeO_2_-10Al_2_O_3_-10CaO-5CaF_2_-5Na_2_O; GGBBN: 65GeO_2_-15Ga_2_O_3_-10BaO-5BaF_2_-5Na_2_O; GGCN: 80GeO_2_-10Ga_2_O_3_-5CaO-5Na_2_O; GGLN: 55GeO_2_-30Ga_2_O_3_-10La_2_O_3_-5Na_2_O; GALN: 70GeO_2_-15Al_2_O_3_-10La_2_O_3_-5Na_2_O, mol.%)

Mechanical resistance of the glass is a key point to manufacture a robust and durable microsystem, in particular for its utilization under high pressure and temperature conditions. In order to evaluate and compare the germanate glasses mechanical behaviours with that of the Pyrex®, measurements of Vickers hardness, Poisson’s ratio, Young’s and Coulomb’s moduli have been realized. Vickers hardness values and photographs of indentation prints obtained are presented in Figure 5.

The Vickers hardness values measured for the five germanate glasses (Figure 5) are lower than the Pyrex® value, which is 634 kgf.mm^−2^. The GGLN and GALN samples have hardness values of 609 kgf.mm^−2^ (4% lower than Pyrex®) and 578 kgf.mm^−2^ (9% lower than Pyrex®), respectively. However, the hardness values obtained for the GGLN and GALN glasses are superior to those measured for the GACCN, GGBBN and GGCN glasses.

The elastic modulus (E and G) and Poisson ratio (υ) obtained from the USE method and density measurement are listed in Table 4.

**Table 4.** Density ρ, Young’s modulus E, Coulomb’s modulus G and Poisson ratio υ obtained by ultrasonic echography (USE) at ambient temperature and pressure for the different glasses studied

Glass sample	ρ [±0.005 g.cm^−3^]	E [±0.1 GPa]	G [±0.1 GPa]	υ [±0.01]
Pyrex®	2.228	64.1	27.7	0.16
GACCN	3.625	72.0	28.5	0.26
GGBBN	4.383	64.0	25.0	0.28
GGCN	3.914	63.6	25.5	0.25
GGLN	4.205	90.6	34.6	0.31
GALN	4.238	80.8	31.4	0.29

First, the GGBBN and GGCN samples have elastic modulus values slightly lower than those of Pyrex® glass. Indeed, the Coulomb modulus obtained are 25.0 and 25.5 GPa for the GGBBN and GGCN, respectively, while it is 27.7 GPa for the Pyrex®. The Young’s modulus values for these three samples are very closed to each other (about 64 GPa) given the accuracy of measurement (± 0.1 GPa). These relatively low Young’s modulus values can be explained by a larger free volume and more unreticulated vitreous networks, despite the 5% of Na_2_O which should slightly increase the elastic modulus, as it has been already demonstrated for Na_2_O concentration up to 20 mol.% in the GeO_2_-Na_2_O and GeO_2_-PbO-Na_2_O glassy systems [57,58]. This phenomenon is explained by an increase in the compactness of the glassy network for Na_2_O concentration between 0 and 20 mol.%. Beyond 20 mol.%, the depolymerization of the germanate network reduces the compactness, so the Young’s modulus decrease.

Then, the elastic moduli for the GACCN, GGLN and GALN samples are higher than those of the Pyrex®. The GGLN and GALN samples possess the highest Young’s moduli which are 90.6 and 80.8 GPa, respectively. These values are explained by the addition of high field intensity cations (Al^3+^ and Ga^3+^) in larger proportion, which increases the average binding energy density, and thus the Young’s modulus values. The Poisson ratio for these two glasses is also higher than that of the Pyrex®, indicating that the crosslinking of the germanate vitreous network is lower than the silicate network in the Pyrex® glass. Therefore, these germanate glasses are more subject to deformations under compression or stretching if compared to the Pyrex®. This can induce a higher probability of cracks' appearance during the anodic bonding, because the glass is compressed by the copper cathode (see Figure 2). From these results, the GGLN and GALN glasses have overall the best resistance to external physical aggressions among the studied glasses. The GALN glass, which was also determined as the most promising germanate glass with respect to its optical, thermal and electrical properties, has thus been selected to perform the anodic bonding tests.

**Figure 6.**
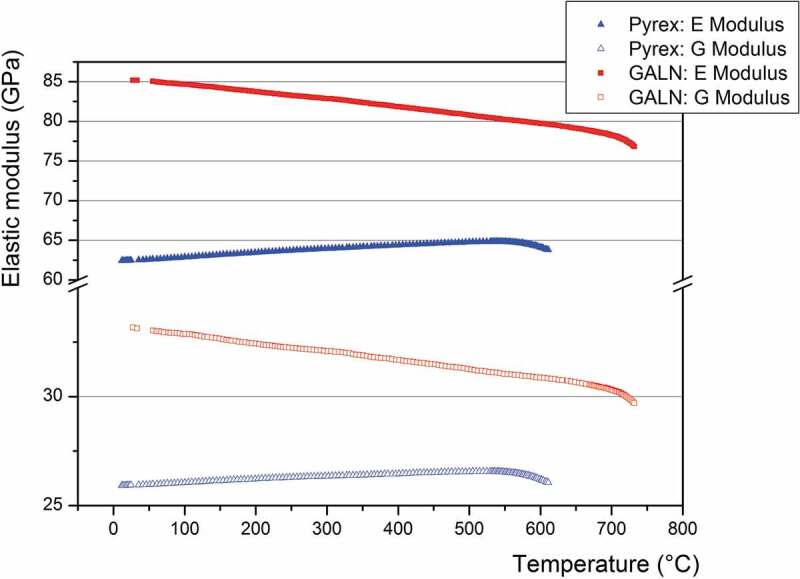
Evolution of elastic modulus obtained by RFDA as a function of the temperature for the Pyrex® and the 70GeO_2_-15Al_2_O_3_-10La_2_O_3_-5Na_2_O (GALN) samples

Besides, it is important to know the elasticity of the material as a function of temperature for the development of the anodic bonding process with the germanate glass, but also for the future utilization of the microsystem produced that will be subjected to temperature variations. The evolution of the elastic modulus as a function of temperature (measured by EDFA) is presented in Figure 6 for the GALN and Pyrex® glasses. One can remind that both glasses exhibit similar electrical conductivities from room temperature to 500°C.

First, one can see in Figure 6 that the elastic modulus of the GALN and Pyrex® glasses behaves differently according to the temperature. Indeed, both the E and G moduli of the Pyrex increase with increasing the temperature, as already reported in [59], while they decrease for the GALN glass. Beyond the glass transition temperature, the elastic modulus for the two compositions starts to collapse because of the glass transition from the isotropic elastic solid state to the viscoelastic state. However, the elastic modulus remains higher for the GALN glass in all the temperature range from room temperature to 740°C. The Coulomb moduli G of the Pyrex® are ranging from 26.0 to 26.6 GPa between 20°C and 560°C (corresponding to the glass T_g_), while for the GALN glass they are ranging from 33.2 to 30.5 GPa between 30°C and 690°C (corresponding to the glass T_g_). The same behaviour is observed for the Young moduli E, which vary from 62.6 to 64.9 GPa between 20°C and 560°C for the Pyrex®, and from 85.4 to 78.5 GPa between 30°C and 690°C for the GALN glass. Note that these values are consistent with those measured by ultrasound, considering the accuracy measurement. In summary, despite a lower Vickers hardness and a higher Poisson’s ratio measured on the GALN glass if compared to the Pyrex®, the alumino-germanate glass generally exhibits superior mechanical strength under temperature variations compared to the other glasses studied in this work.

### 3.2. Germanate glass anodic bonding on silicon wafer

**Figure 7.**
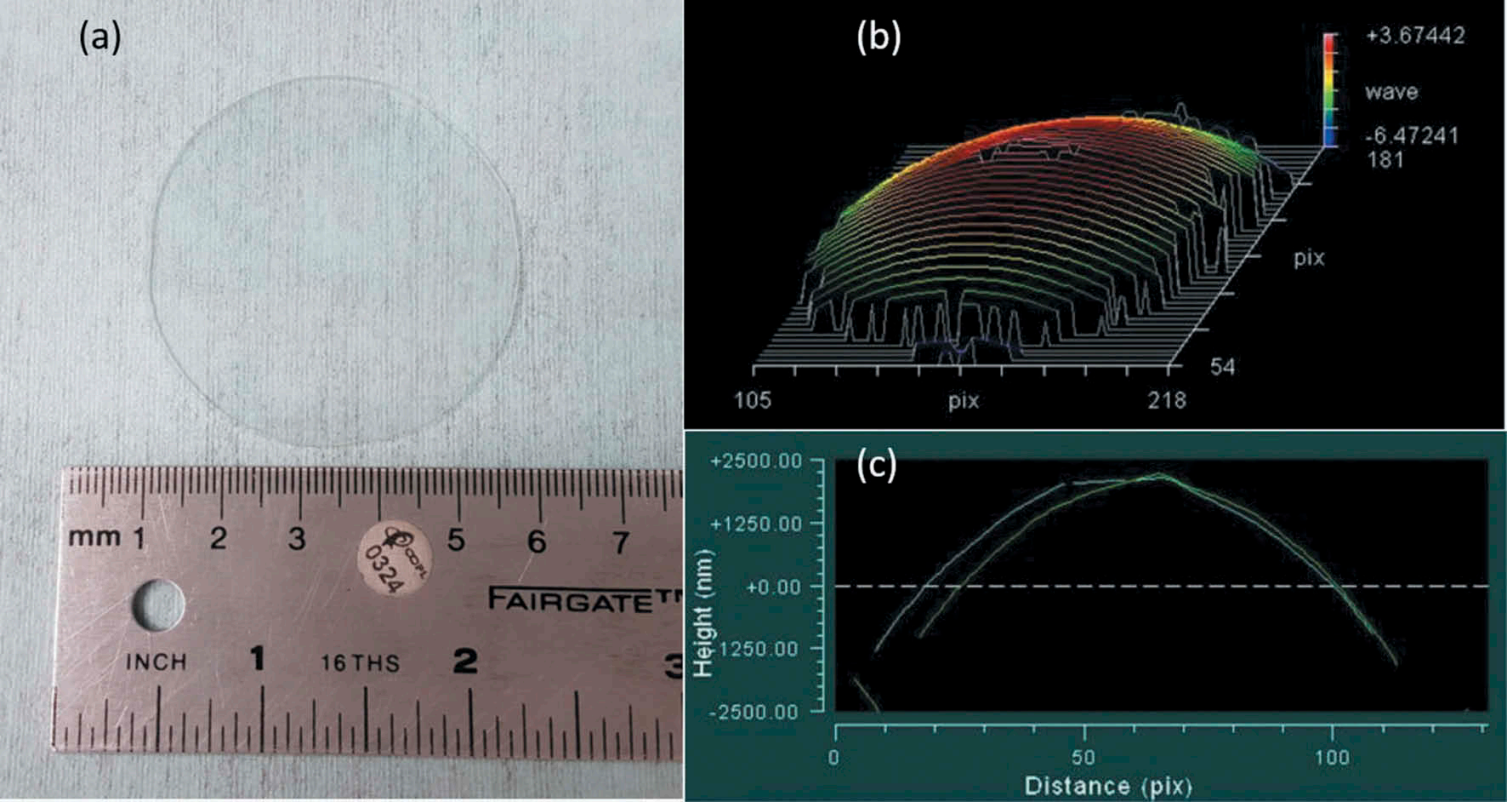
GALN germanate glass plate (5 cm diameter and 1 mm thickness) used for anodic bonding tests: (a) Photograph of the plate. (b) 3D sample surface reconstructed after optical surface state interferometry measurement. (c) 2D surface profile crossing the sample centre. Distance unit in pix represents 0.05 cm/1 pix

An example of a polished GALN germanate glass plate 5 cm diameter and 1 mm thickness is presented in Figure 7(a). The samples present a slightly concave form, as measured with the optical surface state interferometer, with a roundish surface of ~3 µm between the centre and the edge of the sample (Figure 7(b,c)). The result obtained for the commercial Pyrex® plate of the same dimensions is ~1 µm.

**Figure 8.**
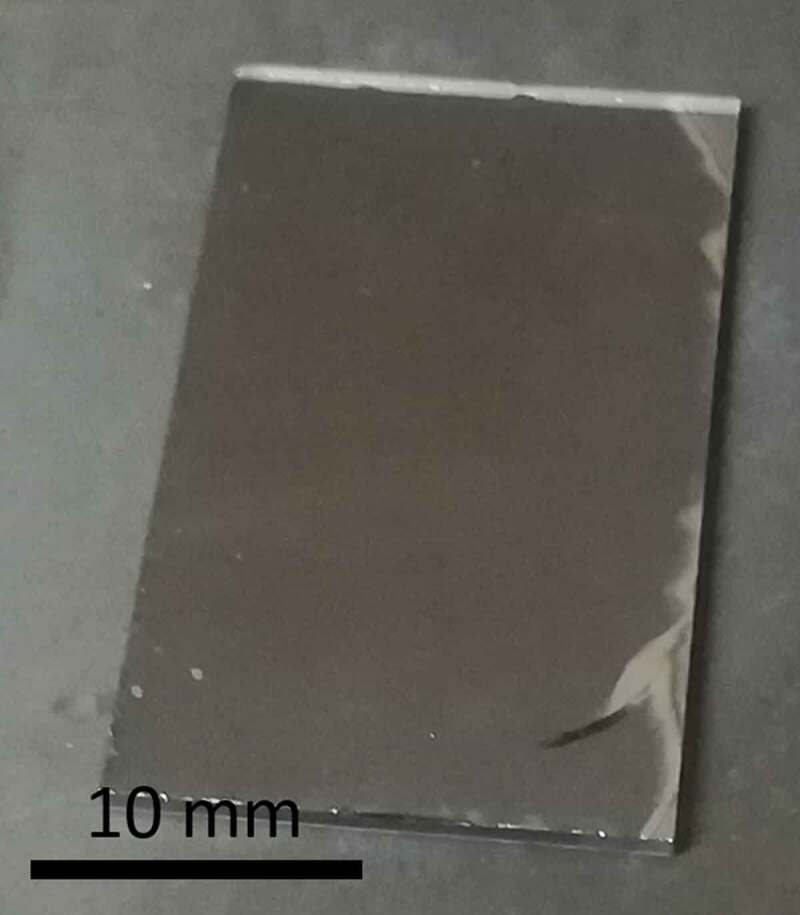
Photograph of a bonded assembly made of 70GeO_2_-15Al_2_O_3_-10La_2_O_3_-5Na_2_O (GALN) glass and silicon wafer

As illustrated in Figure 2, the bonding principle is based on the migration of Na^+^ and O^2-^ ions between two electrodes. High temperatures and voltages are applied during the bonding process to improve the mobility of the ions in the glass. More details about the anodic bonding are given elsewhere in the literature [18,60,61]. The main interest of anodic bonding compared to glass fusion bonding is that the temperature is generally much lower than T_g_, thus preventing from channel deformation in case of small features. As mentioned in the experimental section, 50 mm-diameter and 1 mm-thick plates of germanate glass (GALN) have been prepared to perform the anodic bonding tests. The efficiency of the implemented experimental procedure has been first validated by the successfully bonding of commercial Pyrex® plates onto silicon wafers. Then, after slight adjustments of temperature and duration, GALN glass substrates have been bonded to silicon wafers, as illustrated in Figure 8.

As seen in Figure 8, no bubbles or dust are observed at the interface between both materials, showing the efficacy of the cleaning and bonding procedures implemented. This result demonstrates, therefore, the feasibility of bonding a mid-IR transparent germanate glass to a silicon wafer anodic bonding technique. However, it can be noticed that some small regions on the right border of the assembly did not bond along with the appearance of a crack, due to constraint formation during cooling. This material stress is generated by the bonding process as expected because of the slightly larger TEC difference between silicon and GALN glass (2.6x10^−6^ K^−1^ vs 6.9 × 10^−6^ K^−1^, respectively) compared to silicon and Pyrex® glass (2.6x10^−6^ K^−1^ vs 3.3 × 10^−6^ K^−1^, respectively). Work is in progress to minimize the formation of stress across the germanate-based glass/Si assembly during the treatment. First, the glass TEC will be further decreased by adjusting its chemical composition. Then, conditions as heating and cooling rates, bonding temperature and applied voltage *vs* time will be adjusted along the process according to chemical composition changes.

**Figure 9.**
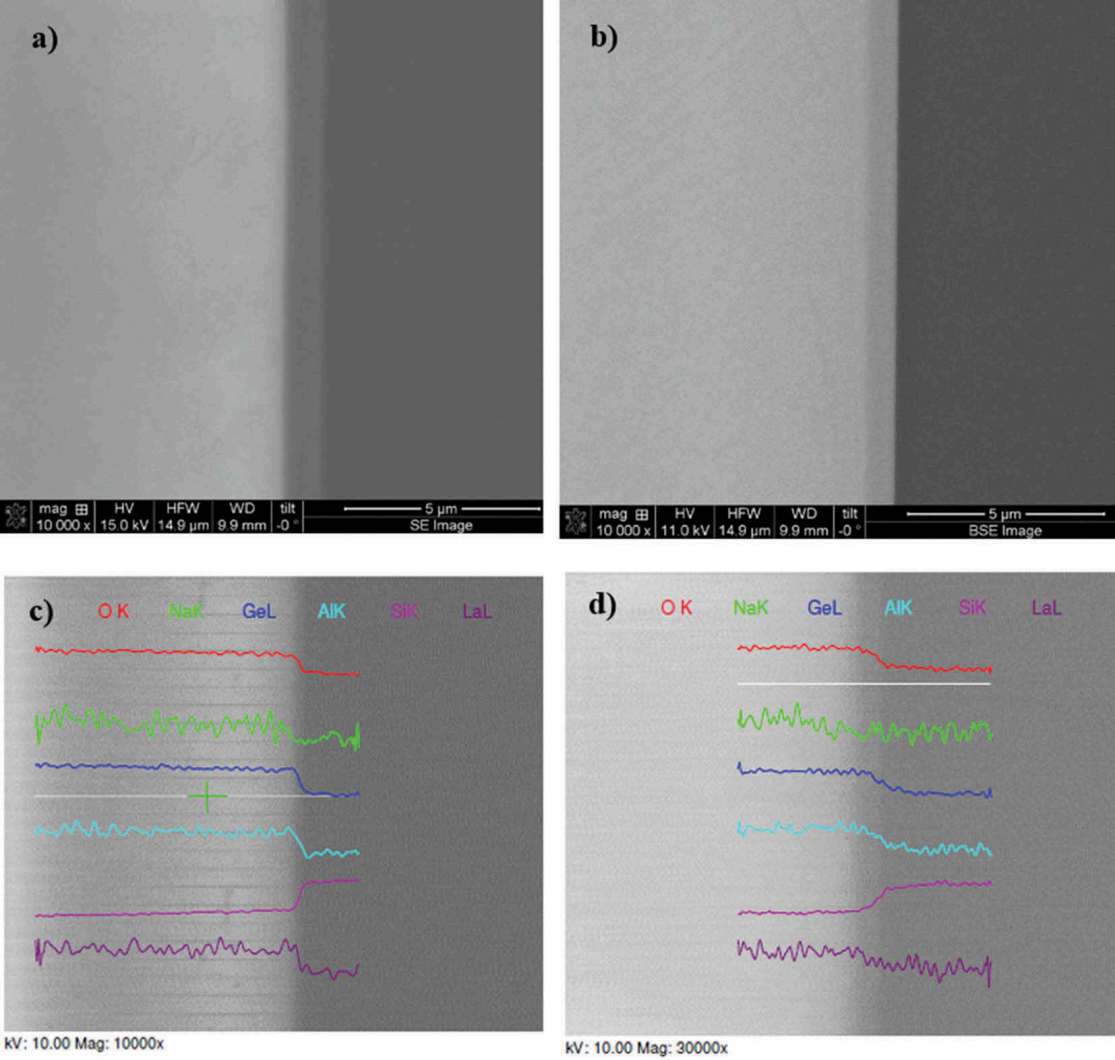
SEM images showing the interface (cross-section) of bonded germanate GALN – silicon wafer in secondary electron (SE) mode (a) and backscattered electron (BSE) mode (b). Dark region corresponds to silicon whereas the clear one is the glass. EDAX elemental chemical analysis profiles recorded for each along a line (in white) crossing the interface, at different magnification rates (c-d)

Scanning electron microscopy analyses have been performed on the obtained GALN–Si assemblies to investigate the interface. SEM micrographs obtained in secondary electrons and backscattered electron modes are presented in Figure 9. A transition region of about 400–500 nm width is clearly observed at the interface on all SEM images, between the GALN glass (clear region) and the silicon wafer (dark region). This suggests a modified chemical composition in this specific region, if compared to the glass and the wafer.

The concentration profile of each chemical element has been extracted from the elemental chemical analyses recorded by EDAX along lines crossing the interface, as shown in Figure 9. First, one clearly observes the abrupt decrease of oxygen, germanium, aluminium and lanthanum at the interface, from the glass to the Si wafer. Meanwhile, an abrupt increase in the silicon content is observed, as expected. One can also notice the better signal over noise ratio of data recorded for oxygen, germanium and silicon elements, owing to their larger content vs sodium, aluminium and lanthanum. These decrease/increase of elemental contents match perfectly with the transition regions above mentioned, suggesting that element migration is confined within this region. The situation for sodium cations is however less clear. Although their migration from the interface region is expected, i.e. a depletion area at the glass/Si interface, it is difficult from the data presented here to conclude, essentially because of its lower concentration. Nonetheless, from the data recorded at a higher magnification rate, it seems that the decrease of sodium content is not occurring within the transition region as for other elements, but slightly before. This tends to confirm the existence of a sodium-depleted region at the glass/Si interface.

## 4. Conclusions

Mid-IR transparent soda-germanate glasses belonging to five different vitreous systems (GeO_2_-Al_2_O_3_-CaO-CaF_2_-Na_2_O; GeO_2_-Ga_2_O_3_-BaO-BaF_2_-Na_2_O; GeO_2_-Ga_2_O_3_-CaO-Na_2_O; GeO_2_-Ga_2_O_3_-La_2_O_3_-Na_2_O; and GeO_2_-Al_2_O_3_-La_2_O_3_-Na_2_O, mol.%) have been investigated for their potential to be coupled with silicon wafers by anodic bonding process to replace the Pyrex® glass substrate in high pressure/high-temperature microreactors. The optical, thermal, mechanical and electrical properties of the prepared glasses have been systematically studied and compared to those of the Pyrex®, permitting to determine the best candidate for the targeted application: 70GeO_2_-15Al_2_O_3_-10La_2_O_3_-5Na_2_O (mol.%). An appropriate cleaning procedure has been then implemented for the 50 mm-diameter glass plates produced and the silicon wafers. The anodic bonding parameters leading to a successful germanate glass bonding to the silicon wafer have been determined. Although issues related to material stress still have to be addressed, the work presented here is a promising proof-of-concept demonstrating the potential of fabricating new microfluidic HP/HT systems displaying transparency from the near UV to the mid-IR for *in situ* spectroscopy.
